# From Myricetin to the Discovery of Novel Natural Human ENPP1 Inhibitors: A Virtual Screening, Molecular Docking, Molecular Dynamics Simulation, and MM/GBSA Study

**DOI:** 10.3390/molecules27196175

**Published:** 2022-09-21

**Authors:** Shaohan Song, Zhiyu Shao

**Affiliations:** 1 Shanghai Foreign Language School Affiliated to Shanghai International Studies University, Shanghai 200083, China; 2 College of Chemistry and Chemical Engineering, Donghua University, Shanghai 201620, China

**Keywords:** ENPP1 inhibitor, virtual screening, biological evaluation, myricetin, molecular docking, molecular dynamics

## Abstract

It was recently revealed that naturally occurring myricetin can inhibit ectonucleotidase ectonucleotide pyrophosphatase/phosphodiesterase 1 (ENPP1), which, in turn, can treat ischemic cardiac injury. However, due to myricetin’s poor druggability, its further developments are relatively limited, which necessitates the discovery of novel ENPP1-inhibiting myricetin analogs as alternatives. In this study, the binding model of myricetin with ENPP1 was elucidated by molecular docking and molecular dynamics studies. Subsequently, virtual screening on the self-developed flavonoid natural product database (FNPD), led to the identification of two flavonoid glycosides (Cas No: 1397173-50-0 and 1169835-58-8), as potential ENPP1 inhibitors. Docking scores and MM/GBSA binding energies predicted that they might have higher inhibitory effects than myricetin. This study provides a strong foundation for the future development of ischemic cardiac injury drugs.

## 1. Introduction

The ectonucleotide pyrophosphatase/phosphodiesterase (ENPP) family consists of single-pass transmembrane ectoenzymes [1]. Based on their common phosphodiesterase (PDE) domain, all ENPP members are able to hydrolyze extracellular nucleotides [1]. Each enzyme has clearly established substrate selectivity and catalytic domains that share structural similarities. Angiogenesis [2]; bone mineralization [3,4]; cell proliferation [5] and motility [6]; digestion [7]; and other activities are all impacted by ENPP catalysis. Evidence also supports that non-catalytic functions of ENPPs exist in cell signaling [8].

Among the ENPP family, ENPP1 is a type II membrane-bound glycoprotein that is essential for several physiological functions, such as insulin signaling and bone mineralization [9,10,11]. ENPP1 hydrolyzes pyrophosphate or phosphodiester linkages in a variety of extracellular substances, including nucleotides and lysophospholipids. It is conserved in vertebrates and slows the mineralization process by converting ATP into AMP, and inorganic pyrophosphate by hydrolysis [12]. Additionally, ENPP1 blocks insulin signaling, and insulin resistance is linked to an ENPP1 polymorphism [1,7].

ENPP1 has been found to be crucial for immune cell penetration into tumors [13]. It was discovered that ENPP1 regulates the cyclic GMP-AMP synthase (cGAS)-stimulator of interferon genes (STING) pathway by hydrolyzing 2’,3’-cyclic GMP-AMP (cGAMP). In the tumor microenvironment, it also helps to produce the immunosuppressive signal, adenosine, in lymphoid tissue. The cGAS/STING-driven type I IFN response is attenuated by ENPP1’s hydrolysis of cGAMP [14]. By increasing STING activation and lengthening the half-life of cGAMP in mice that have ENPP1 knocked out, inhibiting ENPP1 has emerged as a promising strategy for cancer immunotherapy [15,16].

**Figure 1:**
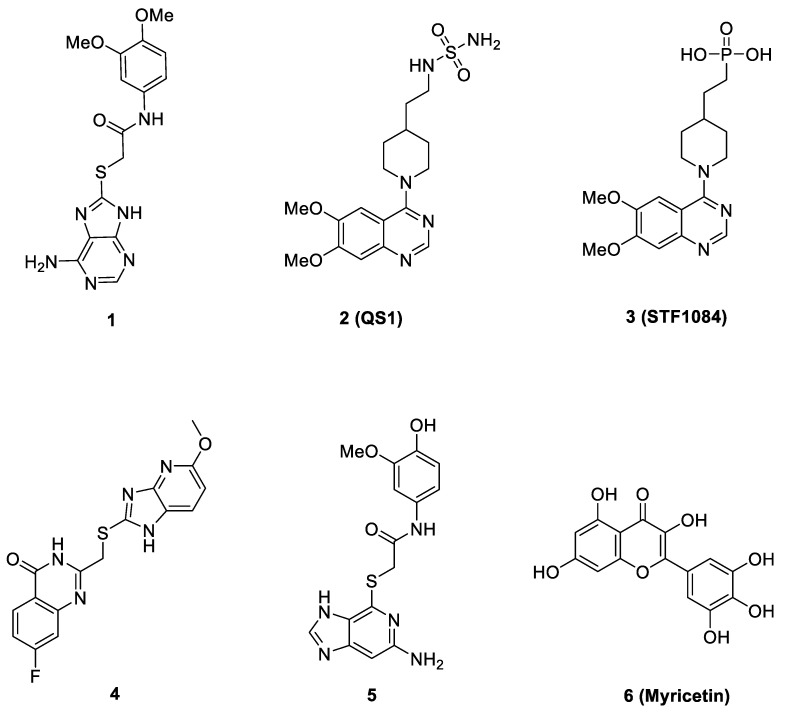
Molecular structures of several representative reported ENPP1 inhibitors.

Only very few non-nucleotide ENPP1 inhibitors (Figure 1) with different scaffolds have been discovered, including thioacetamide inhibitor **1** [17], and quinazoline–piperidine–sulfamide inhibitor **2** (QS1) [18]. Following optimization of compound **2** led to the discovery of a highly potent phosphonate ENPP1 inhibitor (STF-1084, **3**) [15]. Recently Tan et al. reported a novel ENPP1 inhibitor **4**, which demonstrated an IC50 value of 0.188 μM against human ENPP1 at the molecular level. Compound **4** increased the expression levels of IFN-β in vivo, which can be applied to boost antitumor immunity for cancer immunotherapy [19]. Kulkarni et al. recently reported their work on the discovery of a series of promising ENPP1 inhibitors based on a thioguanine scaffold. Their work led to the discovery of compound **5**, which demonstrated to have low sub nM potency and also good efficacy in the LLC1 lung cancer syngeneic mouse model [16].

A more recent study by Li et al. reported that cardiac injury induced the expression of ENPP1 and administration of uridine, or genetic targeting of the ENPP1/AMP pathway enhanced repair, after cardiac injury [20]. In particular, their high-throughput screening (HTS) work led to the identification of myricetin, a small-molecule natural product (**6**, Figure 1), as a potent ENPP1 inhibitor. The administration of myricetin after heart injury rescued pyrimidine biosynthesis in nonmyocyte cells, augmented cardiac repair, and postinfarct heart function [20]. Since some other flavonoid compounds were also reported to elicit anti-myocardial ischemic effects [21,22,23,24,25,26,27,28,29], some other mechanisms including antioxidant and anti-inflammatory activities might also be involved with myricetin [30,31,32]. Novel flavonoid ENPP1 inhibitors might have similar therapeutic potential to rescue cardiac injury and thus are greatly needed. In addition, the binding model of myricetin with its target remains unknown. Thus, to address the demand for rescuing cardiac injury, it is imperative to initiate a further medicinal and chemistry effort. This effort should focus on the discovery of better myricetin-like natural product molecules that are not only potential ENPP1 inhibitors but also have some other common cardiovascular protective benefits, such as in flavonoids.

HTS has become a routine drug discovery process for the identification of novel hit compounds against interesting targets. This process can screen huge chemical libraries rapidly to find the most promising compounds [33,34]. However, it also requires considerable cost and time. As a powerful computational approach to screening large chemical libraries, virtual screening (VS) can save time and costs in the drug development process, which complements HTS effectively in recent years [35,36,37].

In this study, the binding model of myricetin with ENPP1 was firstly elucidated by molecular docking studies. This was further confirmed by molecular dynamics simulations and experimental determination via SAR studies of its structural analogs. Furthermore, based on the model of myricetin binding with ENPP1 and our in-depth understanding of the essential intermolecular interactions from molecular dynamics studies, virtual screening of novel myricetin-like human ENPP1 inhibitors was also performed. This was achieved via an integrated VS workflow to screen the self-developed flavonoid natural product database (FNPD); two flavonoid glycosides were identified as potential ENPP1 inhibitors, and their inhibitory effects were predicted to be superior to that of myricetin when referring to docking scores and MM/GBSA binding energies. This strongly supports future development of new ENPP1 inhibitors for treating ischemic cardiac injury.

## 2. Results

### 2.1. In Vitro ENPP1 Inhibition by Myricetin and pH Does Not Influence Its Activity

**Figure 2:**
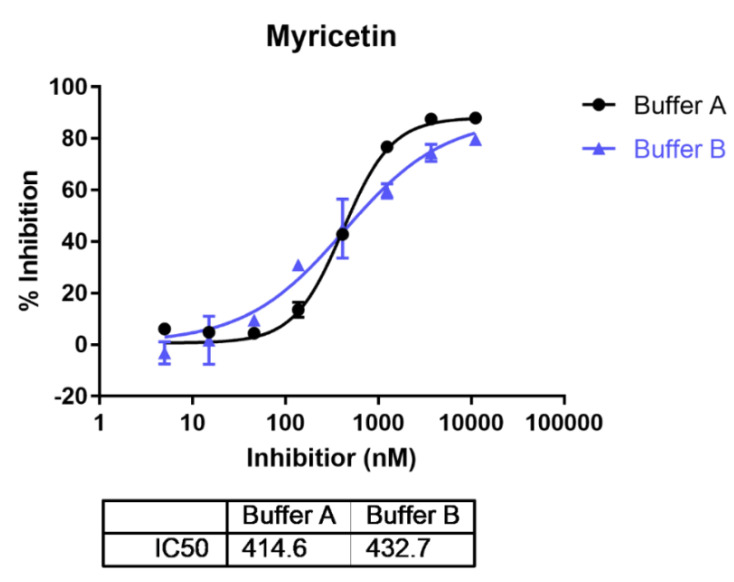
The inhibitory activities for myricetin on ENPP1 assayed under different pH conditions. (Buffer A: pH 9.5; Buffer B: pH 7.4).

Previous studies found that the activity of ENPP1 showed metal ion dependency and pH dependency [38]. ENPP1 showed higher activity at higher pH with the addition of Ca^2+^ and Zn^2+^. Myricetin was reported to inhibit human ENPP1 activity with an IC50 of 4.8 μM in an HTS study [20]. However, whether the pH condition influences the inhibitory activity of myricetin, on ENPP1, is still uncertain. We found myricetin showed close IC50s with 414.6 nM and 432.7 nM assayed in buffer A (pH 9.5), and buffer B (pH 7.4) (Figure 2), suggesting that pH does not influence ENPP1 inhibition by myricetin. Hence, myricetin may show unique binding behavior compared to others of the reported ENPP1 inhibitors that demonstrated potent inhibition only under basic conditions [38].

### 2.2. Molecular Docking of Myricetin with ENPP1 and Further Validations

**Figure 3:**
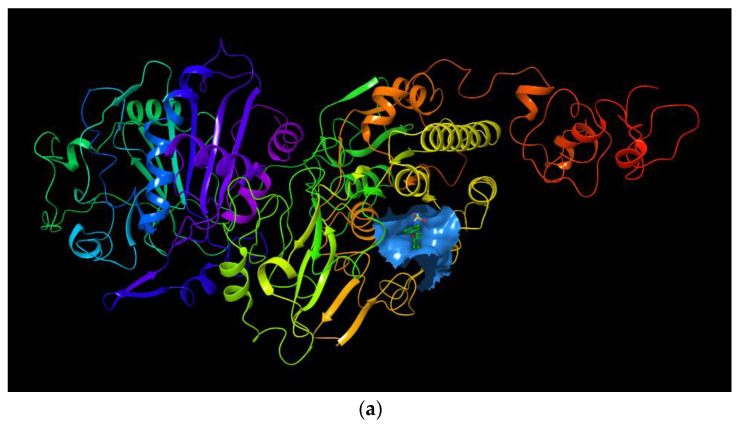

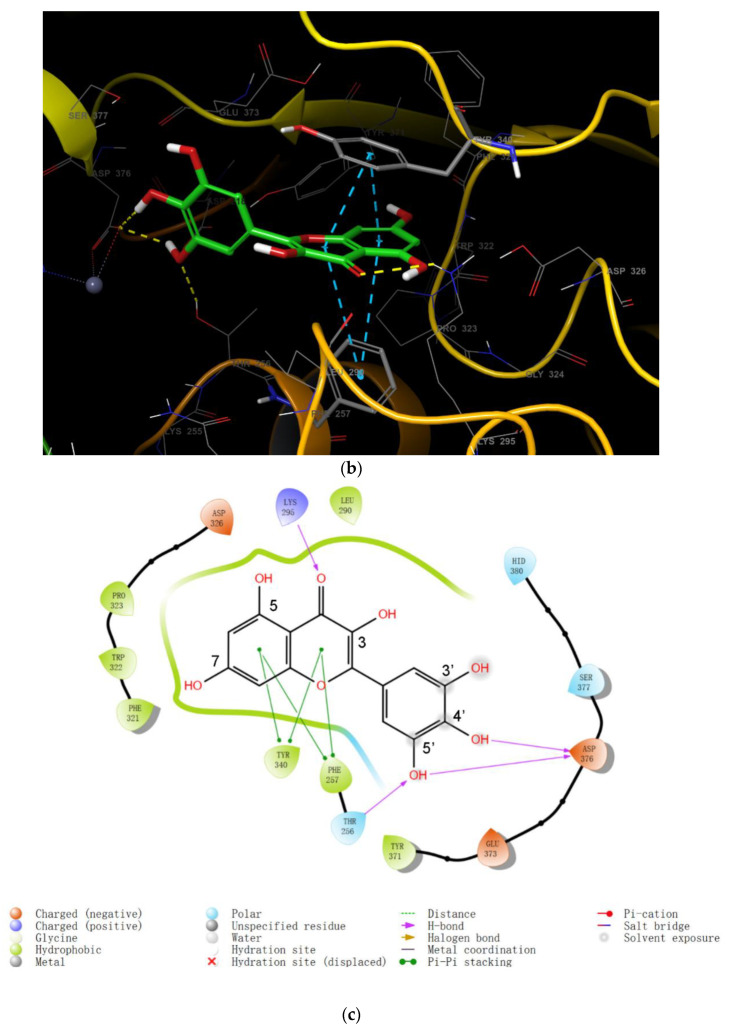
(**a**) The binding region in the entire ENPP1; (**b**) the binding model of myricetin with the ENPP1 from docking study; (**c**) 2D summary of interaction analysis results of ENPP1-myricetin complexes.

The binding region in the entire ENPP1 is shown in Figure 3a. As a positive control molecule, the original ligand in the crystal structure (PDB ID: 6wew) was extracted and docked into the active site of ENPP1. The binding pose was superimposed considerably well with that of the crystal structure complex, and the RMSD value was 0.4945 based on the heavy atoms of the ligand. This methodological investigation indicates that the Glide docking method is applicable to this system.

For myricetin, two main representative binding models were obtained, among which the biggest difference is the benzopyrone scaffold or the 2-phenyl group of myricetin, which form better π-π interactions with the target. Since π-π interaction generally can only be considered via high-level quantum chemical calculations, it is not, therefore, explicitly described in traditional molecular mechanics force fields [39]. It seems reasonable that benzopyrone can form stronger π-π interactions since it is a large, delocalized π system and the withdrawing 4-carbonyl group also helps to increase the π-π interactions with the neighboring Tyr340 or Phe257 amino acid residues. Based on these considerations, and also the satisfactory docking scores, we propose that the binding model in Figure 3b is reasonable for myricetin. In this model, the crystal structure complex was stabilized via π-π interactions and hydrogen bonding interactions. The benzopyrone skeleton of the myricetin molecule plays an important role in the interaction with ENPP1, in which the parent nucleus forms a parallel-displaced π-π stacking interaction with Tyr340 and a T-shape π-π interaction with Phe257 (Figure 3c); 4-oxo forms a hydrogen bond with Lys295; the 3′,4′-dihydroxyl of the 2-phenyl forms two hydrogen bonds with Thr256 and Asp376; and the 7-OH possibly forms a hydrogen bond with Phe321 judging from the distance.

**Figure 4:**
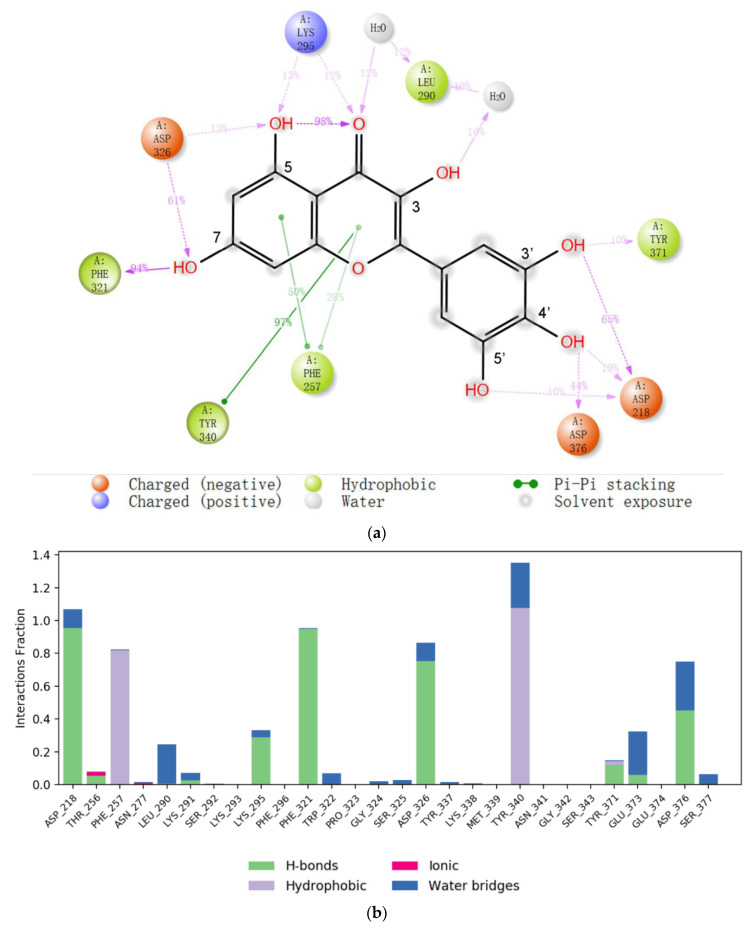
(**a**) The 2D summary of interaction analysis results of ENPP1-myricetin complexes. The interaction pairs that occurred during more than 10% of the simulation time are included. (**b**) Interaction fraction summary of ENPP1–myricetin contacts. Graph is normalized for the total simulation time. Interaction-fraction values over 1.0 indicate that the residue has multiple contact routes for interacting with the ligand.

The reasonability of this binding model of myricetin was further supported by molecular dynamics simulations. For one of the other representative models, in which the 2-phenyl group of myricetin adopts π-π interaction with ENPP1, it underwent relatively big RMS deviations during MD simulations. However, the RMSD of the ENPP1 protein Cα atoms remained stable for our proposed binding model during the whole of the MD simulations, implying the system reached equilibrium at the beginning. There are many more intermolecular forces between the ligand and the protein (Figure 4). Notably, the 5′-hydroxyl could also form a water-mediated hydrogen bond with Asp218 which would maintain for 10% of the simulation time; further, the 3′,4′-dihydroxyl formed a hydrogen bond mainly with Asp218 and Asp376, respectively. Therefore, the 3′,4′,5′-trihydroxyl group also played a very important role in the binding with the target.

**Figure 5:**
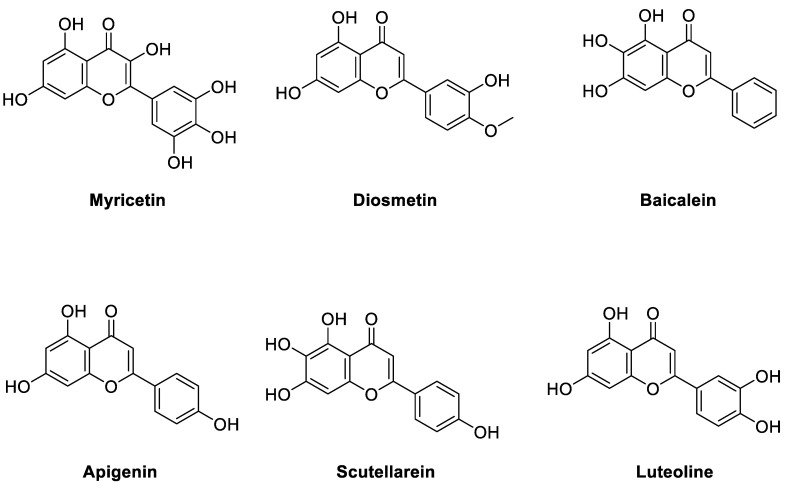
Structures of the SAR studies of myricetin-like flavonoid compounds.

To further validate the binding model, several flavonoid compounds in Figure 5, including diosmetin, baicalein, apigenin, scutellarein, and luteolin, were purchased and determined for their enzyme inhibitory activities. The main differences between the purchased flavone compounds vary in the substitution group of the 2-phenyl group of the flavonoid scaffold. From the MD studies, the 3′,4′,5′-trihydroxyl phenyl group play an important role in binding with the target. It is interesting that from our preliminary biological investigation, luteolin was found to have a small amount of inhibitory activity (32% inhibition at 3.7 uM) while the other compounds, except myricetin, are completely inactive. Clearly, our proposed binding model can effectively explain the SARs of myricetin derivatives. Therefore, the binding model proposed by us was supported by both the theoretical computations and the SAR studies of myricetin derivatives.

### 2.3. Virtual Screening of Novel ENPP1 Inhibitors

**Figure 6:**
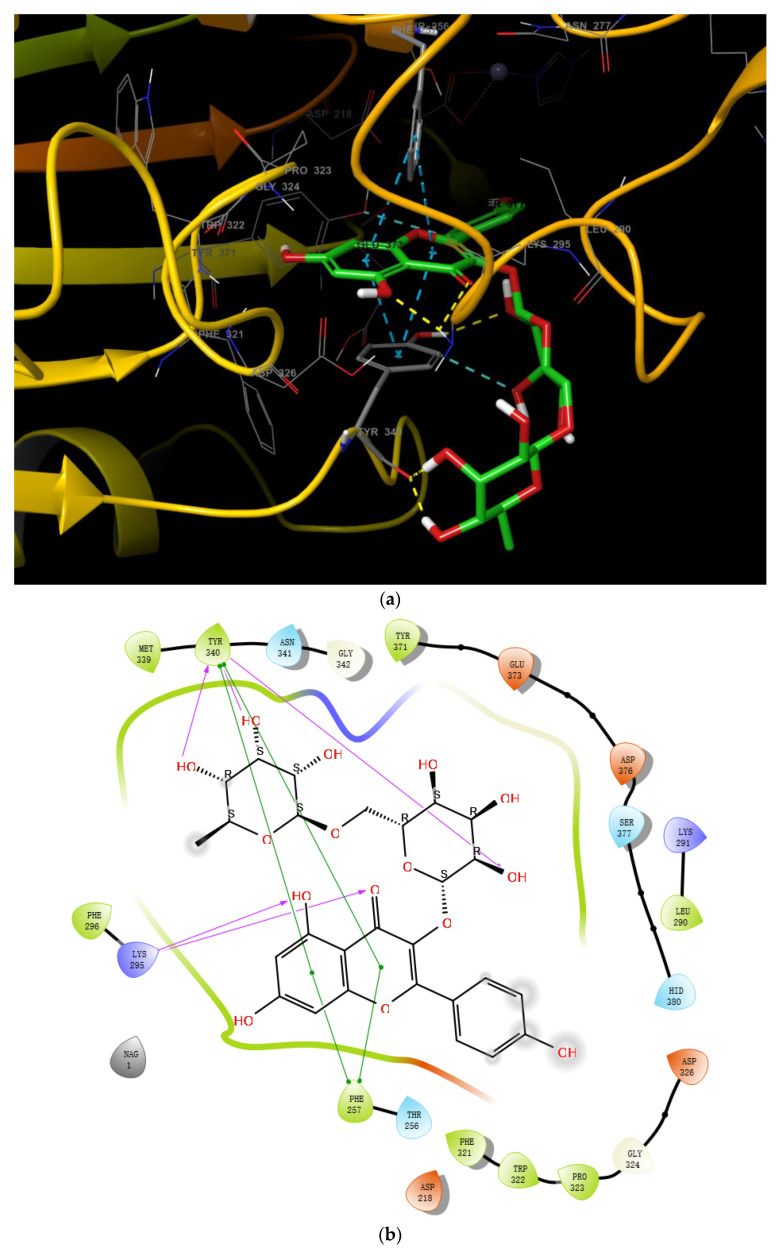

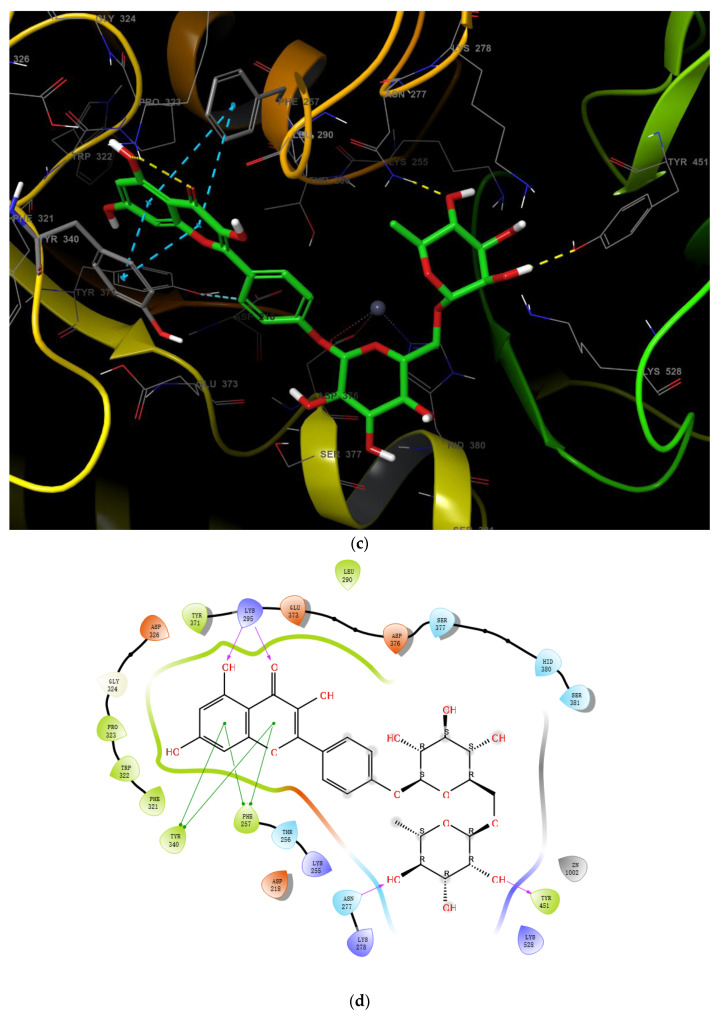
The 3D and 2D binding models of the two flavonoid glycosides identified as potential ENPP1 inhibitors from virtual screening studies. (**a**,**b**) were for Compound **1** (Cas No: 1397173-50-0); (**c**,**d**) were for Compound **2** (Cas No: 1169835-58-8).

Starting from the essential scaffold of 3,5,7-trihydroxy-2-phenyl-4H-chromen-4-one of myricetin for the binding with ENPP1 (identified from computational studies), the substructure search implemented in Scifinder was then employed and 1886 flavonoid molecules were retrieved and exported to SDF file format. The 1886 molecules were further processed, and the resulting 5690 models including tautomeric, stereochemical, and ionization variations were then submitted to virtual screening studies. In the first HTVS run, 793 binding poses were obtained. In the second SP docking run, 720 poses were obtained. In the XP run, 151 binding poses were obtained. After MM/GBSA calculations, two flavonoid glycosides (Cas No: 1397173-50-0 and 1169835-58-8), Table 1) were identified as potential ENPP1 inhibitors. Based on better docking scores and MM/GBSA binding energies, their inhibitory effects were predicted to be superior to that of myricetin. These flavonoid aglycones share very similar binding orientations with myricetin, and the disaccharides formed additional hydrogen bonds with the target (Figure 6). Notably, hydrophobic interactions were also formed between the disaccharides and the protein. This strongly warrants future biological studies and might provide better medicines for ischemic cardiac injury.

## 3. Discussion

Myricetin is a naturally occurring flavonoid antioxidant, which is found in vegetables, fruits, tea, and wine. This valuable dietary constituent has shown promising therapeutic potential, including anticancer, antidiabetic, osteoporosis protection, and anti-inflammatory effects [30,40]. In the case of myocardial dysfunction, myricetin can suppress the inflammatory cytokines and reduce platelet aggregation [32]. Recently it was reported to be an effective ENPP1 inhibitor. Additionally, administration of myricetin after heart injury helps to rescue pyrimidine biosynthesis in nonmyocyte cells, as well as augmented cardiac repair and postinfarct heart function [20]. 

Despite a wide spectrum of pharmacological effects and low toxicity, myricetin’s poor water solubility, bioavailability, and stability limit its further clinical use. Therefore, novel flavonoid compounds, which may show better therapeutic benefits via similar mechanisms, are greatly needed. In this study, two flavonoid glycosides were discovered from virtual screening studies. Generally, higher negative Glide docking score values indicate higher affinity between the receptor and the ligands. However, empirical scoring functions, such as Glide scores, cannot give a reliable prediction. MM/GBSA is much more accurate in binding free energy calculations and has been successfully applied in drug design [41,42,43,44]. The two flavonoid glycosides have shown better docking scores and MM/GBSA binding energies than those of myricetin, implying their ENPP1 inhibitory activities might outweigh that of myricetin. This suggests these two flavonoid glycosides might have considerable potential in the treatment of cardiac injury based on their probable better inhibitory activities on ENPP1.

Due to their complex chemical structures, lack of accessible commercial supply (or expensive prices), these two predicted ENPP1 inhibitors cannot be obtained at this time. Therefore, one limitation of the study is the lack of in vitro enzyme inhibition and in vivo data. Additional efforts, such as isolation from plants or chemical synthesis, are strongly recommended to obtain the two compounds and thus fully characterize their ENPP1 inhibitory activities and also to test their in vivo cardioprotective activities. Notably, the virtual screening methods used in this study will also be helpful to discover novel ENPP1 inhibitors of other chemical skeletons, which may further be used in cancer immunotherapy.

## 4. Materials and Methods

### 4.1. Molecular Docking and Structure-Based Virtual Screening

#### 4.1.1. Receptor Preparation 

The binding model studies of myricetin, and other molecules investigated here, were performed by molecular docking using the Glide software from Schrodinger (released 2021-2). The protein structures of ENPP1 (PDB ID: 6wew [45]) were downloaded from the RCSB website (http://www.rcsb.org, accessed on 20 June 2022). Routinely, protein structure preparation was achieved using the Protein Preparation Wizard, including adding hydrogen, filling in missing side chains, removing crystallographic water molecules, assigning partial charges, etc. It is noted that the phosphate anion coordinating with the essential zinc atoms of the active site, needs to be removed from the crystal structure since it is not necessary for the enzyme to elicit catalytic activity. The complex was minimized in two steps: firstly, only the hydrogen atoms were allowed to move, and secondly, all the heavy atoms were optimized until the root-mean-square deviation (RMSD) reached a maximum value of 0.3 Å. 

#### 4.1.2. Receptor Grid Generation 

The Receptor Grid Generation module in Glide was applied to generate a receptor grid file for docking, in which the binding site was identified using the existing ligand in the crystal structure. 

#### 4.1.3. Preparation of Flavonoid Natural Products Database

Starting from the scaffold of 3,5,7-trihydroxy-2-phenyl-4H-chromen-4-one (which was identified to be essential for the binding with ENPP1 identified from docking studies), the substructure search function implemented in Scifinder was employed and 1886 flavonoid molecules with the scaffold were retrieved and exported to SDF file format. All the 2D molecules were prepared by LigPrep to produce corresponding low-energy 3D structures for further virtual screening studies. LigPrep is a powerful tool that can consider ionization states, tautomers, stereochemistries, and ring conformations. It can generate 32 stereoisomers at most for each input structure. The optimized flavonoid molecules were stored in Mae file format as our self-built Flavonoid Natural Product Database (FNPD). Such a name is used for convenience and does not mean it covers all the reported flavonoid compounds in the literature.

#### 4.1.4. Molecular Docking Studies of Myricetin

The docking accuracy was evaluated with the existing ligand of the crystal structure and used as a positive control molecule for methodological investigation. The positive control molecule and myricetin were docked into the binding site of ENPP1 using the Glide XP mode. The ligands were treated as flexible; all the docked compounds were evaluated by the Glide scoring function, and the binding models with satisfactory docking scores were also analyzed using the MM/GBSA free energy calculations.

#### 4.1.5. Identification of Novel Human ENPP1 Inhibitors by Structure-Based Virtual Screening of FNPD

The virtual screening workflow protocol in the Glide software was used to discover better myricetin-like compounds from our FNPD. The protocol starts from HTVS to SP then to XP, and it enriches the data at each level such that only a few compounds, satisfying some requirements, are submitted to the next highest accuracy level calculations. 

### 4.2. Molecular Dynamics Simulation and Analysis

Molecular Dynamics (MD) simulations were performed using Desmond software. The System Builder in Maestro was used to set up the simulation systems. The ENPP1/ligand complex models were placed in the orthorhombic box at a buffer distance of 10 Å to create a hydration model. An SPC water model was used to create the hydration model [46]. The cut-off radii for van der Waals and electrostatic interactions, time step, and the initial temperature and pressure of the system were set to 9 Å, 2.0 fs, 300 K, and 1.01325 bar, respectively. Additionally, the sampling interval during the simulation was set to 100 ps. Finally, MD simulations were performed under the NPT ensemble for 40 ns using an OPLS4 force field [47]. Interaction analysis between the target and the ligands was performed with The Simulation Interactions Diagram.

### 4.3. In Vitro ENPP1 Enzyme Inhibition Assay 

The assay was performed at 37 ℃ in a total volume of 50 μL in a clear bottom 384-well microplate. The reaction mixture contained 200 μM p-Nph-5′-TMP and the test compound (as per the concentration required) in buffer (consisting of 50 mM Tris, 250 mM NaCl, 500 μM CaCl2, 10 μM ZnCl2) with two pH conditions (Buffer A: pH9.5, Buffer B: pH7.4). The total volume of 50 μL contained 25 μL of the reaction mixture; 5 μL of tested compound (with final DMSO 1% (*v*/*v*)); and 20 μL of hENPP1 (with a final concentration of 1 nM). The reaction was then initiated by the addition of 25 μL of the p-Nph-5-TMP substrate at a final concentration of 0.2 mM, and the reaction mixture was incubated for 30–60 min at 37 °C. The amounts of released p-nitrophenolate were measured at 405 nm. The activity of each compound was compared with the reaction found in the absence of synthesized compounds. Percentage hENPP1 activity was determined, for different concentrations of the test inhibitors, by comparing the absorbance of inhibitors versus blank. The IC50 values were determined by plotting the percent inhibition versus inhibitor concentration curves using a four-parameter non-linear regression curve fit in GraphPad Prism^®^ software.

## 5. Conclusions

Based on the latest report on myricetin’s ENPP1 inhibitory activity and promising in vivo activities in treating cardiac injury, this study was designed to discover novel human ENPP1 inhibitors from natural sources.

In the present study, in vitro ENPP1 inhibitory assay proved the absence of influence via pH conditions on myricetin’s inhibitory activity on ENPP1. Secondly, it was found that the binding model of myricetin with ENPP1 was elucidated through molecular docking, which was further validated by molecular dynamics simulations. In the binding model, the benzopyrone skeleton of myricetin plays an important role in the interaction with ENPP1 via π-π interaction with Phe257 and Tyr340. Furthermore, the 3′,4′,5′-trihydroxyl phenyl group also formed essential hydrogen bond interactions with the target. It is interesting that from our biological results, luteolin was found to have a small amount of inhibitory activity (32% inhibition at 3.7 uM) while the other myricetin analogs are completely inactive, which can be adequately explained by our proposed binding model of myricetin.

In addition, on the basis of the self-developed flavonoid natural products database (FNPD), virtual screening of novel human ENPP1 inhibitors was conducted. The most important and clinically relevant finding was that the two natural flavonoid glycosides were predicted to have strong ENPP1 inhibiting potential, suggesting their expected therapeutic benefits in cardiac injury treatment.

These findings have significant implications for the understanding of myricetin and similar flavonoid compounds. The proposed myricetin binding model with ENPP1 has extended our knowledge of myricetin’s model of action and will be helpful for structural optimization to find novel flavonoid compounds with better ENPP1 inhibition and druggability properties. In particular, the two flavonoid glycosides (Cas No: 1397173-50-0 and 1169835-58-8) identified from the virtual screening study could have even superior activities on ENPP1 and thus this warrants further biological evaluations. In conclusion, this work provides a strong basis for future development of new ENPP1 inhibitors for treating ischemic cardiac injury.

## Figures and Tables

**Table 1:** Docking scores and MM/GBSA scores of two flavonoids predicted as bioactive ENPP1 inhibitors compared with myricetin.

Cas No.	Chemical Structures	Docking Score	MM/GBSA ∆G Bind (kcal/mol)
**1397173-50-0**	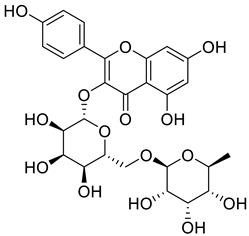	−12.055	−27.18
**1169835-58-8**	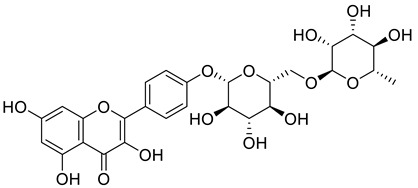	−12.324	−19.73
**529-44-2** (Myricetin)	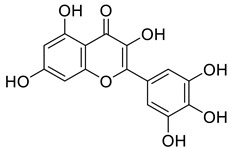	−9.574	−11.19

## Data Availability

Not applicable.
